# Dual-imaging nanoparticles based on surface-modified magnetic nanoparticles and biodegradable photoluminescent polymers

**DOI:** 10.3389/fbioe.2025.1558817

**Published:** 2025-04-10

**Authors:** Nikhil Pandey, Priyanka Iyer, Tejaswi D. Kadapure, Jian Yang, Kytai T. Nguyen, Aniket S. Wadajkar

**Affiliations:** ^1^ Department of Bioengineering, The University of Texas at Arlington, Arlington, TX, United States; ^2^ Department of Biomedical Engineering, The University of Texas Southwestern Medical Center (UTSW), Dallas, TX, United States; ^3^ Department of Neurosurgery, The University of Maryland School of Medicine, Baltimore, MD, United States; ^4^ Marlene and Stewart Greenebaum Comprehensive Cancer Center, The University of Maryland School of Medicine, Baltimore, MD, United States; ^5^ Department of Materials Science and Engineering, Westlake University, Hangzhou, Zhejiang, China; ^6^ Research Centre for Industries of the Future, Westlake University, Hangzhou, Zhejiang, China

**Keywords:** magnetic nanoparticles, core-shell magneto-fluorescent nanoparticles, surface functionalization, theranostic nanoparticles, image-guided drug delivery system, metal-based fluorescence quenching

## Abstract

Theranostic nanoparticles, which combine diagnostic and therapeutic capabilities, have gained significant interest in disease management. We previously developed dual-imaging enabled cancer-targeting nanoparticles (DICT-NPs) composed of a biodegradable photoluminescent polymer (BPLP) and iron oxide-based superparamagnetic nanoparticles (MNPs). While DICT-NPs demonstrated cytocompatibility, magnetic targeting, and imaging capabilities, their fluorescence was inconsistent due to quenching by the MNP core and inefficient BPLP grafting. To address these limitations, we modified the MNP surface with silane, hydroxyapatite, or silane-coupled azide coatings before conjugating with BPLP. The resulting surface-modified DICT-NPs (mDICT-NPs) ranged in size from 200–350 nm and were cyto-compatible with human dermal fibroblasts and normal human prostate epithelial cells. Surface modifications and BPLP conjugation did not affect the superparamagnetic properties of the nanoparticles but enhanced fluorescence by ∼50% compared to the original DICT-NPs. Hydroxyapatite-modified DICT-NPs exhibited significant improvements, including sustained drug release of Paclitaxel and Docetaxel (71% and 68%, respectively, over 21 days), dose-dependent tumor cell uptake in melanoma, thyroid, and prostate cancer cells (with the highest uptake exceeding 60% at 500 μg/mL), and a reduction in cancer cell viability (less than 50% viability in TT thyroid cancer and KAT-4 cancer cell lines). These advancements represent a significant step in overcoming the fluorescence quenching issues associated with iron oxide-based magneto-fluorescent theranostic nanoparticle platforms, enhancing both their imaging and therapeutic potential in cancer treatment.

## Introduction

The ability to image diseased tissues has immense potential for advancing clinical diagnosis and treatments in oncology and fostering advanced biomedical research. Nanoparticle-based imaging agents enable better visualization of tissue structure and physiology, with a modular design allowing for the carrying of multi-modal imaging agents and drugs for target-specific delivery as well as overcoming off-target effects. Such theranostic nanoparticles offer multifunctional capabilities such as monitoring cancer progression, targeting metastases, evaluating treatment responses, and expanding clinical options, which underscores the importance of developing these systems for precision cancer management (Jennings and Long, 2009; Lee and Chen, 2009).

Among theranostic nanoparticle systems, dual-imaging nanoparticle systems have emerged as a promising approach to integrate the strengths of different imaging modalities while mitigating their individual weaknesses. Synergistically integrated dual-modality imaging probes can provide unique biological insights that impact preclinical and, potentially, clinical diagnostics. Such systems take advantage of multimodal imaging properties by incorporating radiolabeled, fluorescent, or magnetic nanoparticles into a single imaging construct, thereby enabling functional and structural characterization *in vivo*. Several dual-modality probes have been developed by combining MRI with optical, nuclear, or ultrasound imaging to enhance spatial resolution, tissue penetration, and diagnostic accuracy. Additionally, several notable reviews provide comprehensive summaries of these dual-imaging nanoprobes for biomedical research (Jennings and Long, 2009; Cheon and Lee, 2008; Kubeil et al., 2022; Lee and Chen, 2009; Duan et al., 2023; Li et al., 2022; Wang et al., 2022; Ong et al., 2021; Smith and Gambhir, 2017; He et al., 2022).

In the realm of dual imaging nanoparticles, Iron oxide-based superparamagnetic nanoparticles (MNPs) are well suited for theranostic applications due to their ability to provide magnetically guided drug delivery, to serve as contrast agents for magnetic resonance imaging (MRI), and to generate hyperthermia for inducing tumor cell death (Sun et al., 2008). Moreover, there remains a dynamic interest in coating MNP surfaces with fluorophores to impart fluorescence dimensionality to complement the MRI capabilities.

To this end, we had previously developed novel dual-imaging cancer-targeting nanoparticles (DICT-NPs) by conjugating biodegradable photoluminescent polymers (BPLPs) to the surfaces of MNPs (Wadajkar et al., 2012; Shan et al., 2018). DICT-NPs had several attractive properties, including magnetic targeting of tumors alongside intrinsic dual-imaging (fluorescence-based optical imaging and MRI) capabilities without relying on exogenous fluorescent organic dyes or quantum dots. However, DICT-NPs exhibited sub-optimal fluorescence in imaging tumor cells after BPLP conjugation to the MNP surfaces. The reduced fluorescence output of the BPLP polymer could be due to interference from the MNP-based core that tends to absorb incidental or emitted light, or from a sub-optimal BPLP coating on the biochemically unaltered surfaces of the MNPs. In addition, the magnetic core of the DICT-NPs may potentially exhibit metal-based quenching of fluorescence from the coated BPLP (Corr et al., 2008). Metals can alter fluorescence properties through interactions with fluorophores, either amplifying or quenching fluorescence depending on the nature of the interaction. This metal concentration-dependent modulation can be useful in biological sensing applications, such as detecting metal ions in solutions (Gai et al., 2012). These theranostic nanoparticles may benefit from amplification of fluorescence; however, the quenching of fluorescence can limit their imaging potential.

**FIGURE 1:**
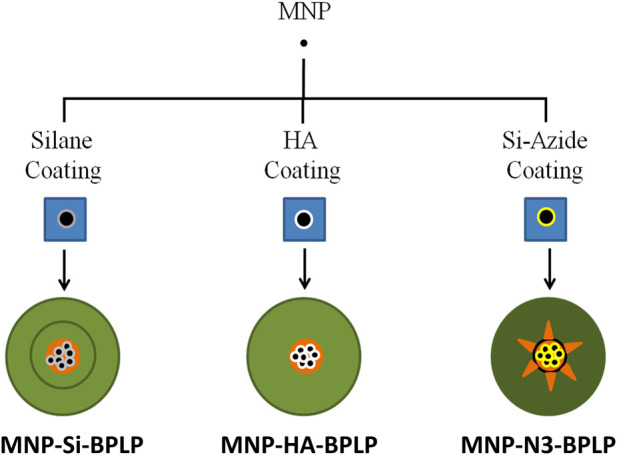
Representation of mDICT-NPs. Biochemical surface modification strategies employed to iron-oxide-based superparamagnetic nanoparticles (MNPs) along with synthesis methodologies to conjugate biodegradable photoluminescent polymers (BPLP) to the surface modified MNPs.

To circumvent the issues of MNP-core-based fluorescence quenching in BPLP fluorescent materials, we biochemically modified the surface of MNPs with the goal: (1) to limit the interaction of the MNP core with the BPLP shell, (2) to provide a biochemically conducive material surface for efficient conjugation of the BPLP shell, and (3) to reduce MNP-core absorption of excitation photons and BPLP fluorescence emission by lowering the absolute absorption cross-section of the MNP-core within the DICT NPs liquid suspensions. We explored the effect of coating silane (Si), hydroxyapatite (HA), and Si-coupled-azide groups (Si-N_3_) on the MNP surface before introducing the fluorescent BPLP shell as a biochemical strategy to mitigate the reduced fluorescence output of DICT NPs, resulting in the development of surface modified DICT-NPs (mDICT-NPs) (Figure 1). To address the sub-optimal fluorescence of DICT-NPs, we employed biochemical surface modification strategies including spatial separation and robust grafting of BPLPs on the MNP surface (Si-N_3_-based mDICT NPs) and using an optically inert coat (HA-based mDICT NPs). Additionally, we achieved compartmentalization of a BPLP polymeric shell and the MNP core through water-oil emulsion methodologies (HA-based mDICT NPs and Si-based mDICT NPs). In this work, the mDICT-NPs were formulated and characterized for physicochemical properties, magnetic and fluorescent properties, non-malignant cell compatibility, *in vitro* effects on tumor cell uptake and viability, and proof-of-concept *in vivo* fluorescence imaging capabilities in ectopic tumor-bearing mice.

## Experimental section

### Materials

All materials were purchased from Sigma-Aldrich (MO) if not specified and were used without further purification or modification. Materials used for experiments included Fe3O4 magnetic nanoparticles (MNPs; Meliorum Technologies, Inc, NY), vinyltrimethoxysilane (VTMS), acetic acid, ethanol-200 proof, potassium phosphate monobasic, calcium nitrate anhydrous, dimethylformamide (DMF) (Sigma Aldrich), copper sulfate pentahydrate, sodium azide, 1,4-dioxane (Sigma Aldrich), poly lactic-co-glycolic acid (Lakeshore Biomaterials, AL), agarose, hydrochloric acid (EMD, MA), potassium thiocynate (Sigma Aldrich), MTS assay kits (Promega, WI), Paclitaxel (LC Laboratories, MA), Docetaxel (Cayman Chemical, MI) and Pico-green DNA assay kits (Promega, WI).

### Cell cultures

Human dermal fibroblasts (HDFα, Invitrogen, CA) were cultured in Dulbecco’s modified eagle medium (DMEM) supplemented with fetal bovine serum (FBS 10% v/v, Atlanta Biologicals, GA) and Penicillin-Streptomycin (PS 1% v/v, Invitrogen, CA). Healthy prostate epithelial cells (PZ-HPV-7, a kind gift from Dr. JT Hsieh, UTSW, Dallas-TX) were cultured in prostate epithelial cell growth medium (PrEGM, Lonza). Prostate cancer cells (PC3, ATCC, VA) were cultured in RPMI-1640 media (Gibco; Invitrogen, CA) supplemented with fetal bovine serum (FBS) (10% v/v) and Penicillin Streptomycin (PS) (1% v/v). Human melanoma cancer cells (G-361) and thyroid cancer cells such as TT and KAT-4 cell lines were obtained from ATCC and cultured in F12K and DMEM media, respectively. All cell cultures were maintained in a standard cell culture incubator and a humid environment at 37°C with 5% CO_2_.

### Surface modification of MNPs

The surface of MNPs (10 nm diameter) was modified with silane (Si), hydroxyapatite (HA), or silane-azide (Si-N_3_). For Si modification, the MNP surface was conjugated with VTMS to form Si modified MNPs (MNP-Si) as described elsewhere (Tran and Webster, 2011; Koppolu et al., 2010). VTMS was selected to covalently bind MNPs, creating a template for BPLP grafting via free radical polymerization (Wadajkar et al., 2012; Sundaresan et al., 2014; Xie et al., 2017; Rahimi et al., 2009).

Briefly, MNPs were dispersed in a mixture (100 mL, 1:99 v/v) of deionized (DI) water and ethanol (Fisher, NJ) by sonication (40 W, 10 min). Subsequently, acetic acid (3 mL, EM Science, NJ) was added, followed by additional sonication (40 W, 10 min). Finally, VTMS (0.49 mL) was added, and the reaction was under magnetically stirred (1,000 rpm) for 24 h at room temperature (Tran and Webster, 2011). The MNP-Si were collected using an external magnet (1.3 T) and washed twice with a mixture of DI water and ethanol (1:99 v/v).

For HA modification, the MNP surface was coated with a hydroxyapatite layer via calcium nitrate (Ca(NO_3_)_2_) and a potassium phosphate (KH2PO4)-based hydrothermal method to form HA modified MNPs (MNP-HA) as described elsewhere (Tran and Webster, 2011). Briefly, a dispersion of Calcium nitrate (Ca(NO_3_)_2)_ (164 mg, 0.10 M) and MNPs (15 mg) in DI water (10 mL) was obtained via sonication (40 W, 10 min). Potassium phosphate (KH_2_PO_4_, 81 mg, 0.06 M) was dissolved in DI water (10 mL) and added dropwise to this dispersion under sonication (40 W, 10 min). Subsequently, the dispersed mixture was treated hydrothermally (200°C, 20 h) to obtain nanocrystalline MNP-HA, which were collected using an external magnet (1.3 T) and washed twice with DI water (Koppolu et al., 2010).

For Si-N_3_ modification, the MNP surface was biochemically altered to introduce azide (N_3_) groups and yield N_3_-modified MNPs (MNP-N_3_), as previously described (Schlossbauer et al., 2008; Kim et al., 2006). Briefly, MNPs (15 mg) were dispersed in DMF (10 mL) via sonication (40 W, 10 min). Next, 3-chloropropyl-trimethoxysilane (CPTES) (100 µL) was added to the above dispersion and the sonication was continued (40 W, 1 h). The resulting silane modified MNPs were collected using an external magnet (1.3 T), washed twice with DI water, and re-suspended in DMF (20 mL). Sodium azide (NaN_3_, 100 µg) was then added to the MNP suspension and stirred overnight at 40°C. Finally, the resulting MNP-N_3_ particles were collected and washed sequentially with DMF, ethanol, and DI water.

### Synthesis of BPLP and BPLP-Alkyne polymers

BPLP was synthesized using 1,8 octane diol, citric acid, and L-cysteine amino acid following our previously developed protocols (Shan et al., 2018; Xie et al., 2017; Xie et al., 2014; Hu et al., 2016; Yang et al., 2009). Briefly, equimolar amounts of citric acid and 1,8 octanediol were mixed, followed by the addition of L-cysteine at a molar ratio of 0.2 relative to citric acid. The mixture was magnetically stirred and melted at 160°C for 20 min. After dissolution, the temperature was lowered to 140°C and the solution was further stirred for 75 min. The obtained oligomers were purified by precipitating them in 1,4 dioxane and lyophilized to obtain BPLP. Similarly, BPLP-alkyne was synthesized with the addition of an alkyne functionalized diol, propargyl 2,2-bis(hydroxymethyl) propionate (PHMP) as a monomer to the citric acid, 1,8 octanediol, and L-cysteine in the reaction mixture (Kim et al., 2006). The BPLP-alkyne polymer was purified using the same processes as described for the BPLP polymer.

### Synthesis of nanoparticles

The unmodified-DICT nanoparticles (BPLP-MNPs) and mDICT NPs were synthesized via a standard single emulsion using our previously developed methods (Wadajkar et al., 2012). Briefly, the BPLP polymer was dissolved in 1,4-dioxane (2.5 mL), and surface-modified MNPs (10 mg) were added and dispersed within via sonication (40 W, 10 min) to form the oil phase. Simultaneously, an aqueous phase of Sodium dodecyl sulfate (SDS) (1.6% w/v, 25 mL) in DI water was prepared. The surface modified-MNP/BPLP oil phase was added in as a drop-wise fashion to the aqueous phase and the resulting dispersion was emulsified via sonication (40W, 5 min) to form the oil in water single emulsion yielding BPLP-MNPs. The BPLP-MNPs were harvested and washed three times with DI water via a 1.3 T external magnet and collected. BPLP-conjugated MNP-N_3_ (MNP-N_3_-BPLP) NPs were synthesized via copper (II) catalyzed click chemistry. Briefly, BPLP-alkyne (200 mg) was dissolved in dimethyl sulfoxide (DMSO, 20 mL), followed by the addition of MNP-N_3_ (15 mg) along with a Cu_2_O microsphere methanol solution (50 μL, 1.25% w/v). The resulting solution was sonicated (40 W, 15 min), followed by incubation at 37°C for 12 h. The resulting particles were collected using an external magnet (1.3 T) and were sequentially washed. The final product was obtained via lyophilization.

### Nanoparticle characterization

We have previously established the physiochemical characterizations of unmodified DICT NPs (BPLP-MNPs) (Wadajkar et al., 2012). For mDICT NPs, the structural morphology was determined using transmission electron microscopy (TEM) (JEOL 1200 EX Electron Microscope). The hydrodynamic diameter and zeta potential of nanoparticles were analyzed using dynamic light scattering (DLS) techniques (ZetaPALS, Brookhaven Instruments, NY). Further, chemical characterization of the nanoparticles material composition was performed using Fourier transform infrared (FTIR) spectroscope (Nicolet-6700, Thermo Fisher Scientific, Madison, WI).

### Magnetic characterization

The amount of iron in the nanoparticles was determined by iron assays, as described previously (Hu et al., 2016). Briefly, either known concentrations of unmodified MNPs only (0–500 μg/mL) or samples of mDICT NPs (0.1% w/v, 100 µL DI water) were dissolved in hydrochloric acid (50% v/v, 5 mL, EMD Chemicals Inc, NJ) at 55°C for 2 h on an orbital shaker. Subsequently, ammonium per-sulfate (50 μg) and potassium thiocyanate (100 μL, 0.1 M) were added in succession, with each addition followed by a 15-min incubation at 55°C on an orbital shaker. The resulting acid-digested samples were transferred to a 96 well plate and analyzed for absorption (λ_max_: 520 nm) using a UV-Vis spectrophotometer (Tecan Ltd, NC). The recorded absorbance values were plotted against MNP standard concentrations to obtain a weight-absorbance standard curve, which was used to estimate the percentage of iron mass present in mDICT NPs. Furthermore, magnetic properties such as saturation magnetization, remanence, and coercivity of the nanoparticles were assessed using a vibrating sample magnetometer (VSM, Weistron, CA). The nanoparticle samples were embedded in wax, mounted on a transparent non-magnetic rod, and a magnetic field was then applied enabling recording of magnetic hysteresis loops for mDICT NPs as well as unmodified MNPs.

### Magnetic resonance imaging

MRI on mDICT-NPs was performed in agarose phantom gels using our previously developed protocols (Wadajkar et al., 2012). Briefly, MR images were obtained using a Varian unity INOVA 4.7 T 40 cm horizontal MR system equipped with actively shielded gradients (Varian, CA) (205 mm with 22 G cm^-1^). The tissue mimicking agarose-based gels were prepared by dissolving agarose (1% w/v) in warm DI water. The mDICT-NPs were dispersed within the agarose solution at 0.1% w/v. Unmodified MNPs, BPLP only and BPLP-N_3_ polymer only, were included as control groups at equivalent concentrations. The nanoparticle samples in agarose gels were placed into a 35 mm volume radiofrequency coil (Varian unity INOVA 4.7T 40 cm horizontal MR machine). Multislice T2-weighted images (TR = 2000 msec; TE = 15 msec; field of view of 30 mm × 30 mm; matrix = 128 × 128; slice thickness = 2 mm) were acquired using a spin echo pulse sequence. The acquired MR images were analyzed in Matlab (Mathworks Inc., Natick, MA) to calculate percentage drops in the MR signal intensities of the T2-weighted images of samples relative to controls.

### Fluorescence imaging studies

Fluorescence imaging of the nanoparticles was performed on an enhanced optical microscope (CytoViva Inc, AL). Nanoparticle suspensions (0.2% w/v) in DI water were drop casted on optical glass slides and images were captured at ×100 magnification. In addition, the fluorescence spectra from the nanoparticles were collected using a fluorometer (SHIMADZU, RF-5301PC) and the excitation/emission maxima were determined using a slit width of 5 nm × 5 nm.

For fluorescence imaging of nanoparticle-cancer cell uptake, 30,000 prostate cancer cells (PC3 or LNCaP) were seeded in a 48 well plate in their respective media as described in the cell culture materials section and allowed to attach and become confluent. Nanoparticles were suspended (100 μg/mL) in the respective cell culture media, added to the cancer cells, and incubated for 2 h at 37°C in a standard cell culture incubator. Subsequently, the media was aspirated, and cell containing wells were washed twice with PBS to remove suspended or unbound particles. Finally, cancer cells were fixed using 4% paraformaldehyde and were imaged for fluorescence under an enhanced optical microscope (Nikon, Nikon Instruments Inc., Melville, NY) using a ×10 objective.

For *in vivo* fluorescence imaging of nanoparticles, the studies were conducted in compliance with the animal welfare policy set forth by the Institutional Animal Care and Use Committee (IACUC), and protocols approved by the University of Southwestern Medical Center at Dallas and the University of Texas at Arlington. Briefly, 6–8 week old NOD SCID nude mice were anesthetized via an isoflurane-based method and PC3 tumors were inoculated subcutaneously into the flanks by injecting 1.5 x 10^6^ PC3 cells. Once the tumors reached 100 mm^3^ in volumetric size, nanoparticles were injected intra-tumorally. After nanoparticle administration, the mice were euthanized via CO_2_-induced asphyxiation and were imaged for fluorescence via the KODAK FX Pro Rodent Imaging System (KODAK, NY).

### *In Vitro* cyto-compatibility

Cytocompatibility of mDICT NPs was evaluated with HDFα and PZ-HPV-7 cells. Briefly, 5,000 cells/well were seeded in a 96 well plate and incubated to facilitate cell attachment and confluence. Varying concentrations (50, 100, 200, 300, 500 μg/mL) of nanoparticles in DMEM media (10% v/v FBS, 1% v/v PS) were added to the cells for 24 h. The cells were then washed thrice with 1x PBS and cell viability was determined using MTS assays (Cell Titer 96^®^ Aqueous One Solution Cell Proliferation Assay, Promega) as per the manufacturer’s instructions.

### *In Vitro* cellular uptake studies

For nanoparticle-cancer cell uptake analyses, 5,000 cells (G3-61, TT, Kat-4, PC3 or LNCaP) were seeded in a 96 well plate in their respective media and allowed to attach for 24 h. Nanoparticles were suspended (0–500 μg/mL) in the respective cell culture media, added to the cancer cells and incubated for 2 h at 37°C in a standard cell culture incubator. Subsequently, the media was aspirated, and cells were washed twice with PBS to remove suspended or unbound nanoparticles. The cells were then lysed using 1X Triton, and the cell lysate sample was used to quantify the total amount of iron as well as the total DNA via iron assays and Pico green DNA assays respectively, following the manufacturer’s instructions. The total iron was normalized to the total DNA within each nanoparticle-concentration group to estimate cellular uptake of nanoparticles.

### Nanoparticle drug loading and release study

Paclitaxel and Docetaxel, as model anti-cancer drugs, were encapsulated in the HA-based mDICT NPs at the time of the double emulsion synthesis via incorporating the drug molecules into the BPLP oil phase. To calculate drug loading efficiency, drug-loaded mDICT NPs (1 mg) were suspended in DCM (1 mL) and the extracted drugs were separated from the MNPs using a 1.5 T externally applied magnet. The supernatant containing drug molecules was analyzed via UV-Vis spectrophotometry (Paclitaxel λ_max_: 230 nm and Docetaxel λ_max_: 235 nm) and the % loading efficiency (L.E %) was calculated using the equation below.

$$\text{Loading Efficiency }\left(L.E\right)\;\left(\%\right)=\left[\;\left[\frac{Initial\;amount\;ofdrug\;used-Amountofdrug\;in\;supernatent}{Initial\;amount\;of\;drug\;used}\right]\times 100\right]$$

Drug release studies were conducted by suspending drug-loaded nanoparticles (10 mg) in PBS (1 mL, pH 7.4) and incubating them at 37°C up to 4 weeks. At each predetermined time point (1, 3, 12, 18, 24, 48, 96, 168, 336, 504 h) of drug release sampling, the nanoparticles were separated via a 1.5 T magnet and the supernatant containing the released drug was collected and analyzed via UV-Vis spectrophotometry to quantify the amount of drugs released at each respective time point. The individual time-point-released drug amounts were summated over the entire 4 weeks of drug release sampling to construct cumulative drug release curves for Docetaxel and Paclitaxel.

### *In Vitro* therapeutic study

Therapeutic effects of drug-loaded mDICT NPs were studied on TT thyroid cancer and KAT-4 cancer cell lines. Briefly, the cells were seeded on 96 well plates at 5,000 cells/well density and were allowed to attach for 24 h. Paclitaxel (PTX) only, unloaded nanoparticles and drug-loaded nanoparticles (1, 10, 50, µg/ml PTX equivalents) suspended in culture media were added and incubated with the cells. After 24 h of incubation, cell viabilities were measured using MTS assays following the manufacturer’s recommended instructions to determine the effects of drug-loaded mDICT NPs on killing cancer cells over the time period.

### Statistical analysis

Results were analyzed using ANOVA with *post hoc* comparisons and t-tests with *P* < 0.05. The sample size was four for all the studies except magnetic characterization by VSM and MRI. The results were presented as mean ± standard deviation.

## Results and discussion

### Nanoparticle physiochemical characterization

For nanoparticle physiochemical characterizations, FTIR was chosen for chemical validation, as it effectively confirms the presence of key functional groups associated with silane, hydroxyapatite, and polymer coatings, providing sufficient evidence of successful surface modifications. TEM was used for structural confirmation, allowing direct visualization of the core-shell architecture and validating the successful modification of the magnetic core with polymer coatings at the morphological level. For size determination, DLS was selected as it measures the hydrodynamic diameter of nanoparticles in solution, offering a more biologically relevant representation of their behavior under physiological conditions. Together, these techniques provide a comprehensive assessment aligned with our study’s focus on fluorescence enhancement and dual-imaging capabilities instead of a detailed elemental quantification and higher-magnification TEM-based structural analysis.

**FIGURE 2:**
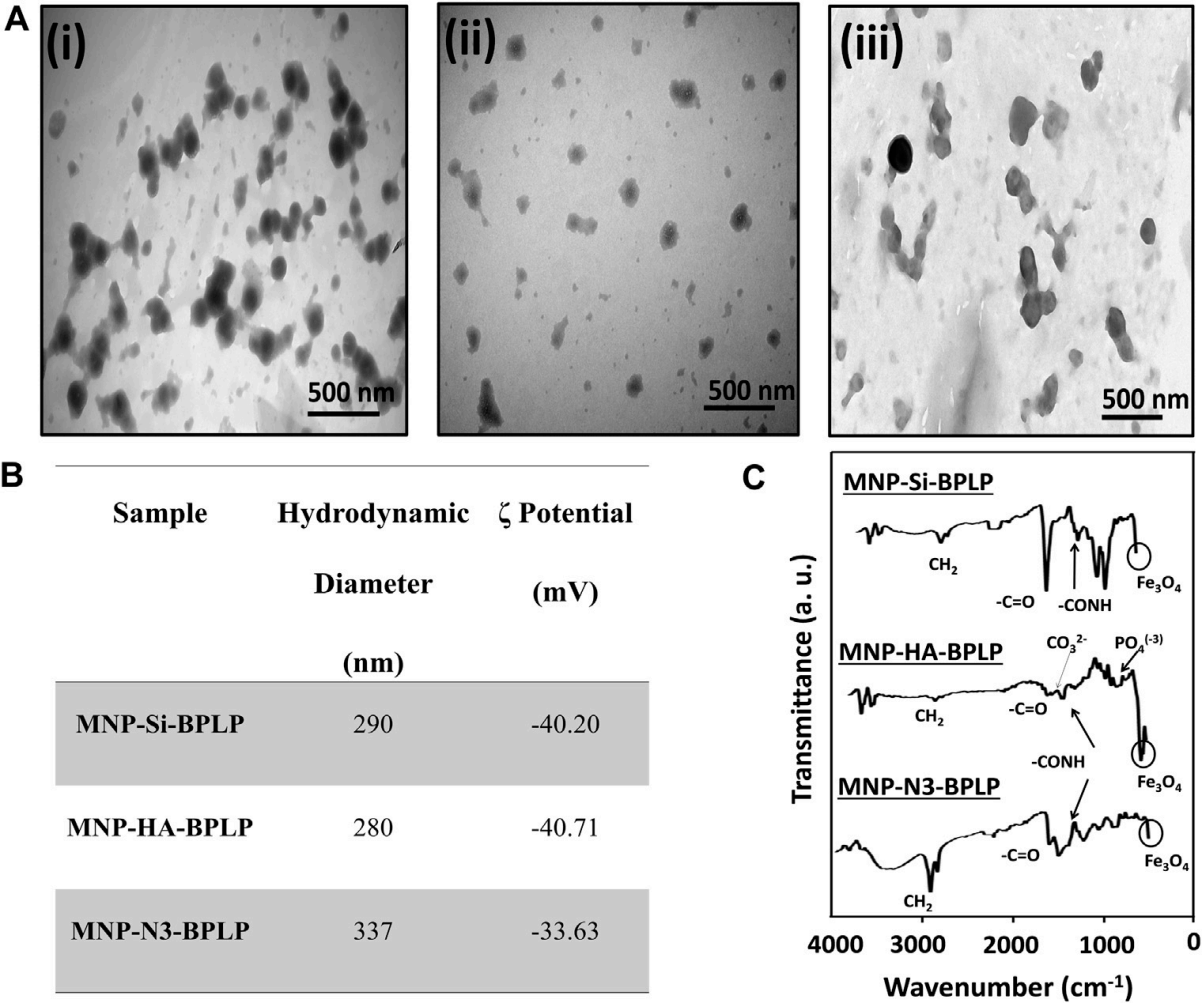
Physiochemical characterization of mDICT-NPs. **(A)** TEM images showing the core-shell structures of NPs: **(I)** MNP-Si-BPLP (ii) MNP-HA-BPLP (iii) MNP-N_3_-BPLP. **(B)** Hydrodynamic diameters and zeta potentials (ζ) of MNP-Si-BPLP, MNP-HA-BPLP and MNP-N_3_-BPLP. **(C)** FTIR spectrums of MNP-Si-BPLP, MNP-HA-BPLP and MNP-N_3_-BPLP showing MNP and BPLP constituent chemical peaks.

The mDICT-NPs are composed of a superparamagnetic iron oxide nanoparticle core with distinct surface modifications (Si, HA, Si-N_3_) to enable differential grafting of the biodegradable fluorescent polymeric shell. The DLS analysis of mDICT-NPs showed a hydrodynamic size within 250–350 nm and zeta potentials within −30 to −40 mV (Figure 2B). TEM imaging further revealed successful assembly of mDICT-NPs and confirmed their MNP core-polymeric shell morphology (Figure 2A). The mDICT-NPs were within the size range (<400 nm) appropriate for retention within the vascular compartment upon intravenous administration and permeate tumor sites through leaky vasculatures via the enhanced permeation and retention (EPR) effect (Alexis et al., 2008; Hobbs et al., 1998). Additionally, the negative surface charges of the mDICT-NPs may potentially repel negatively charged serum albumin molecules, preventing agglutination mediated aggregation (Figure 2B).

The incorporation of the polymeric shell onto the different surface-modified MNPs (Si, HA and Si-N_3_) was confirmed by FTIR analysis (Figure 2C). FTIR analysis was performed to confirm the sequential surface modifications of mDICT NPs. In the synthesis process, HA or N_3_ was first coated onto the MNP surface, followed by BPLP conjugation. As a result, HA-BPLP and N_3_-BPLP do not exist as standalone materials, and separate FTIR controls for these were not included. Instead, FTIR spectra were recorded at each modification step to verify the presence of characteristic functional groups, ensuring successful incorporation of HA, N_3_, and BPLP. This approach provided sufficient chemical validation of the stepwise functionalization process, aligning with the study’s focus on fluorescence enhancement and dual-imaging capabilities. The FTIR spectra of all mDICT-NPs exhibited peaks corresponding to CH_2_ (3,017–3,020 cm^-1^), CO-NH (1,630–1,635 cm^-1^) and citric acid based -C=O (1700–1705 cm^-1^) bonds associated with the BPLP, in addition to the Fe_3_O_4_ peak (540 cm^-1^) associated with the MNPs.

### Magnetic properties

**FIGURE 3:**
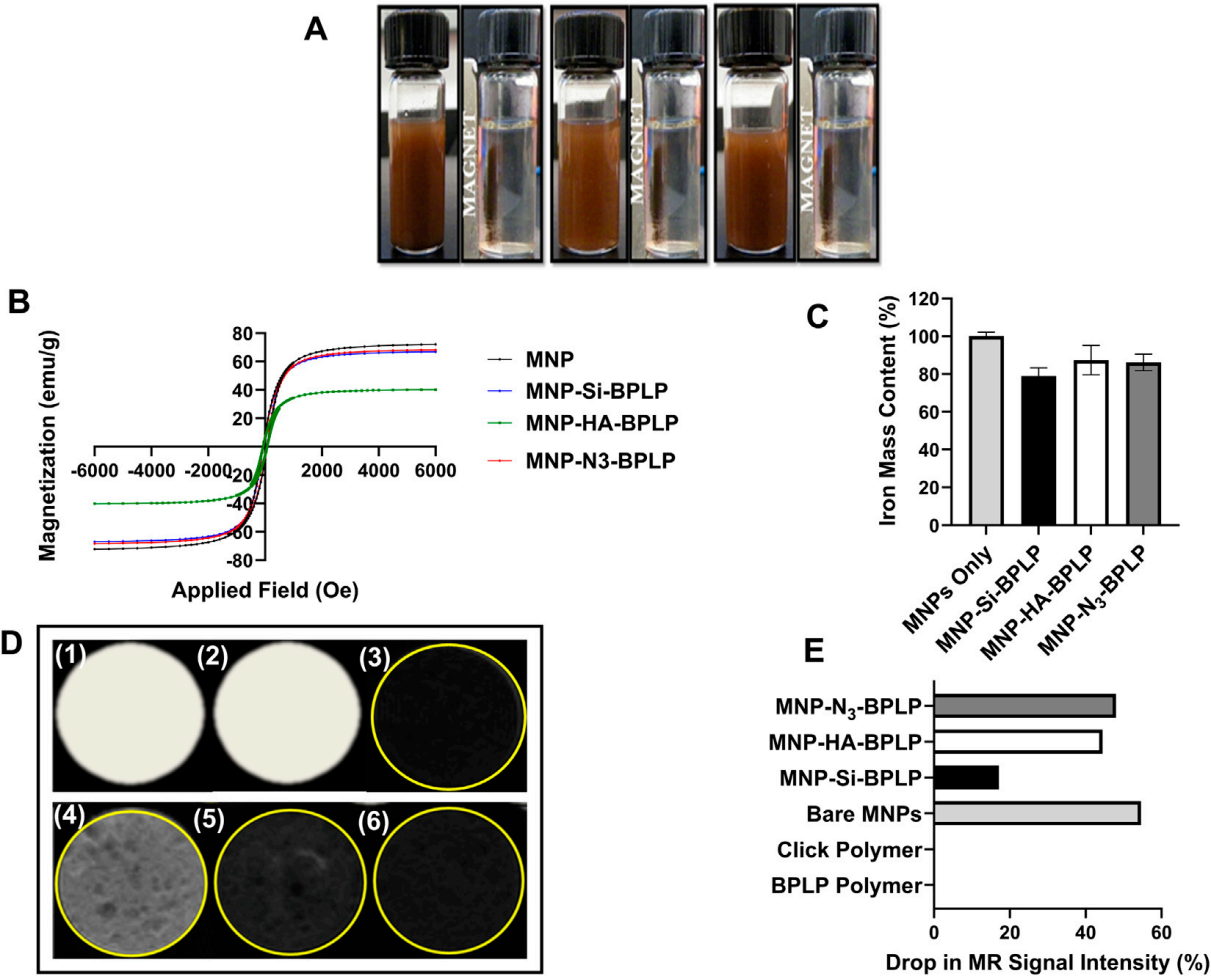
Magnetic characterization of mDICT nanoparticles. **(A)** Magnetic suspension of MNP-Si-BPLP, MNP-HA-BPLP and MNP-N_3_-BPLP particles showing even suspension and active magnetic recruiting upon an application of a 1.5 T bar magnet. **(B)** Hysteresis loops showing retention of superparamagnetic properties of the mDICT nanoparticles. **(C)** Iron mass contents of mDICT NPs. **(D)** T2-weighted MR images of agarose phantom embedded mDICT NPs: (1) BPLP polymer only, (2) BPLP-Alkyne polymer, (3) MNPs only, (4) MNP-Si-BPLP particles, (5) MNP-HA-BPLP particles, (6) MNP-N_3_-BPLP particles. **(E)** MR signal intensity drop from mDICT NPs in comparison to the control groups.

The MNP core of mDICT NPs enables magnetically guided delivery to tissue sites, functions as negative contrast in MRI, and can potentially be utilized as a hyperthermia agent for tumor therapy (Yallapu et al., 2011; Thiesen and Jordan, 2008; Hayashi et al., 2013; Boyer et al., 2010; El-Boubbou, 2018). MNPs typically need surface coatings to improve their biocompatibility, prevent aggregation and impart functionalization sites for further bioconjugation (Ramimoghadam et al., 2015; Favela-Camacho et al., 2016; Xu et al., 2011; Gupta and Gupta, 2005). However, such non-magnetic coating materials can interact with surface atoms of the MNP core resulting in a magnetically disordered surface layer that lowers the total magnetic phase, altering magnetization, hyperthermia generation and MRI-contrast generating properties (Yuan et al., 2012; Millan et al., 2007). To verify mDICT NPs retention of magnetic properties and to investigate T2-weighted MRI contrast generating profiles upon BPLP layer deposition, we conducted vibrating sample magnetometry (VSM) analyses, *in vitro* agarose phantom-based MRI imaging, and quantified the total iron content within each mDICT NP system via spectrophotometry-based iron content assays (Figure 3). Overall, all groups of mDICT NPs showcased superparamagnetic properties were well dispersed in aqueous-based mediums with no signs of agglomeration and could be magnetically recruited to the site of an externally concentrated magnetic field generated via a 1.3 T bar magnet (Figure 3A).

VSM analyses revealed no remanence or coercivity in the hysteresis curves of mDICT NPs (Figure 3B) confirming the retention of superparamagnetic properties of the MNP core. The presence of BPLPs onto MNP-Si or MNP-N_3_ did not substantially alter the total saturation magnetization of these NPs relative to uncoated MNPs (∼65 emu/g). However, MNP-HA-BPLP showed a marked decrease in saturation magnetization values (∼30 emu/g) indicating reduction in the total magnetic phase. Several studies have reported a decrease in saturation magnetization of MNPs when coated with HA, attributing this effect to a decrease in mass ratio of iron oxide within the polymeric-MNP nanoparticle-construct (Wu et al., 2019; Zară-Dănceanu, 2023; Soleymani et al., 2020). In general, within core-shell-MNP nanoparticle systems, the crystallinity and size of the MNP core can dictate the total saturation magnetization of the polymeric MNP nanoparticle-construct (Laurent et al., 2008). It is well-established that deposition of polymeric layers on MNP surfaces typically leads to reduced saturation magnetization of the MNP-polymer nanoparticle-construct via the introduction of non-magnetic surface layers (Gaharwar et al., 2009; Gómez-Lopera et al., 2001; Voit et al., 2001; Sen and Bruce, 2009). Furthermore, it has also been reported that MNP-bio-polymeric composite systems made using water in oil emulsion methodologies that encapsulate MNPs showcase drastic reduction in saturation magnetization (Zhi et al., 2006), possibly due to the presence of large non-magnetic polymeric layers.

Spectrophotometry-based iron content assays reveal no significant differences in the total iron content of mDICT NPs relative to unmodified MNPs (Figure 3C). Since the mDICT NPs possessing HA and silane-based surface modifications have the BPLP polymeric shell incorporated via water in oil-based emulsion methodologies, we attribute the reduction in saturation magnetization values of HA-based mDICT NPs to the presence of a non-magnetic polymeric layer on the surface of MNPs composed of the inorganic-HA layer along with the polymeric BPLP coating.

Recent studies using layer-by-layer methodologies to modify MNP surfaces with polymers also highlight a reduction in saturated magnetization due to the presence of multiple polymeric layers (Akbar et al., 2019). However, controlled modulation of the layer-by-layer processes towards depositing a thin HA-based-polymeric coat onto the MNP surface resulted in retention of their MNP-based saturation magnetization profiles (Lachowicz et al., 2017). These studies and our findings suggest the importance of controlling the thickness of deposited non-magnetic layers on the MNP surfaces.

### Magnetic resonance imaging

The MNP-based core of mDICT NPs via its superparamagnetic nature should affect a reduction in the T2 relaxation time of excited protons, resulting in a decrease in MR signal intensity in T2-weighted MRI images, producing a dark or negative contrast in areas where mDICT NPs localize. It is essential to consider the effect of the biochemical nature of the non-magnetic polymeric coating on the reduction of the magnetic phase of core MNPs. In addition, the polymer coating can also dictate other magnetic properties depending on the size of the coating and the magnetic core such as T2-based relaxation.

To evaluate the MRI-imaging characteristics, we conducted MRI assessments of mDICT NPs in agarose phantoms, a well-established *ex vivo* approach that strongly correlates with *in vivo* MRI assessments (Sattarahmady et al., 2015; Ansari et al., 2021; Melancon et al., 2011). Studies have shown that relaxivity (r_2_ and r_2_*) measurements in agarose phantoms effectively predict nanoparticle performance *in vivo*, as demonstrated by various magnetic nanoparticle formulations (Sattarahmady et al., 2015; Ansari et al., 2021). Additionally, contrast enhancement observed in agarose phantoms is often mirrored in *in vivo* conditions, with superparamagnetic nanoparticles consistently providing significant T_2_-weighted MRI contrast in both settings (Melancon et al., 2011; Liu et al., 2022). Furthermore, nanoparticle behavior, including uptake and distribution, can be assessed in phantoms and serves as a reliable indicator of their *in vivo* performance (Rivière et al., 2005; Billotey et al., 2003). These findings support the use of agarose phantoms as a valid model for evaluating the MRI characteristics of mDICT NPs.

Our *in vitro* agarose phantom-based MRI imaging analyses to simulate accumulation of mDICT NPs within tissues and organs revealed that all mDICT NPs retained the MNP-core-based property to generate a T2-based negative MR contrast despite their surface modifications and presence of a BPLP shell (Figures 3D, E). Notably, agarose phantom-embedded Si-based mDICT NPs had the lowest drop in T2-based MRI signal intensities (Figure 3E), indicating a lengthening of T2 relaxation times resulting in brighter contrast relative to N_3_ and HA based-mDICT NPs. Our iron content analyses indicate that the Si-based mDICT NPs have lower MNP contents (∼10% lower) relative to N_3_ and HA-based mDICT NPs, which could be the reason for their extended T2-relaxation times and the corresponding significantly lower drops in MR intensities, thus appearing brighter in MR contrast.

### Fluorescence imaging

The BPLP shell of mDICT NPs enables fluorescence-based optical imaging, supplementing the MNP core-based MRI imaging mode to facilitate multimodal imaging of cells, tissues, and tumors. One of the major challenges in developing fluorescent polymer-coated-MNP core-shell type of dual imaging nanoparticle systems is controlling the MNP-core mediated fluorescence quenching of the polymeric shell. The interactions between the iron oxide-based MNP core and the proximate surface fluorophores can lead to non-radiative energy transfers, resulting in quenching of fluorescence (Chance et al., 1978; Dubertret et al., 2001). This has been shown for several types of fluorophore entities conjugated to the surfaces of MNPs as well as other metal-based nanoparticle systems (Bertorelle et al., 2006; Qu et al., 2011; Nan et al., 2019; Dulkeith et al., 2002; Anger et al., 2006; N'Guyen et al., 2013). To mitigate such metal-based fluorescence quenching, various bio-chemical strategies have been adopted towards modifying the surface of MNPs to introduce spacer layers separating the fluorescent entity and MNP core. These include (1) providing the MNP with an optically inert and stable shell before introducing the fluorescent entity; or (2) first bio-chemically tethering a molecular spacer to the fluorescent entity thus disjoining it from the MNP core. Overall, studies attempting to prevent MNP surface-mediated quenching of fluorescence have typically focused on silica-based coatings on the MNP surface due to its bio-inertness and optical transparency (Lu et al., 2002; Chekina et al., 2011; Kumar et al., 2008; Ha et al., 2009; Peng et al., 2011).

**FIGURE 4:**
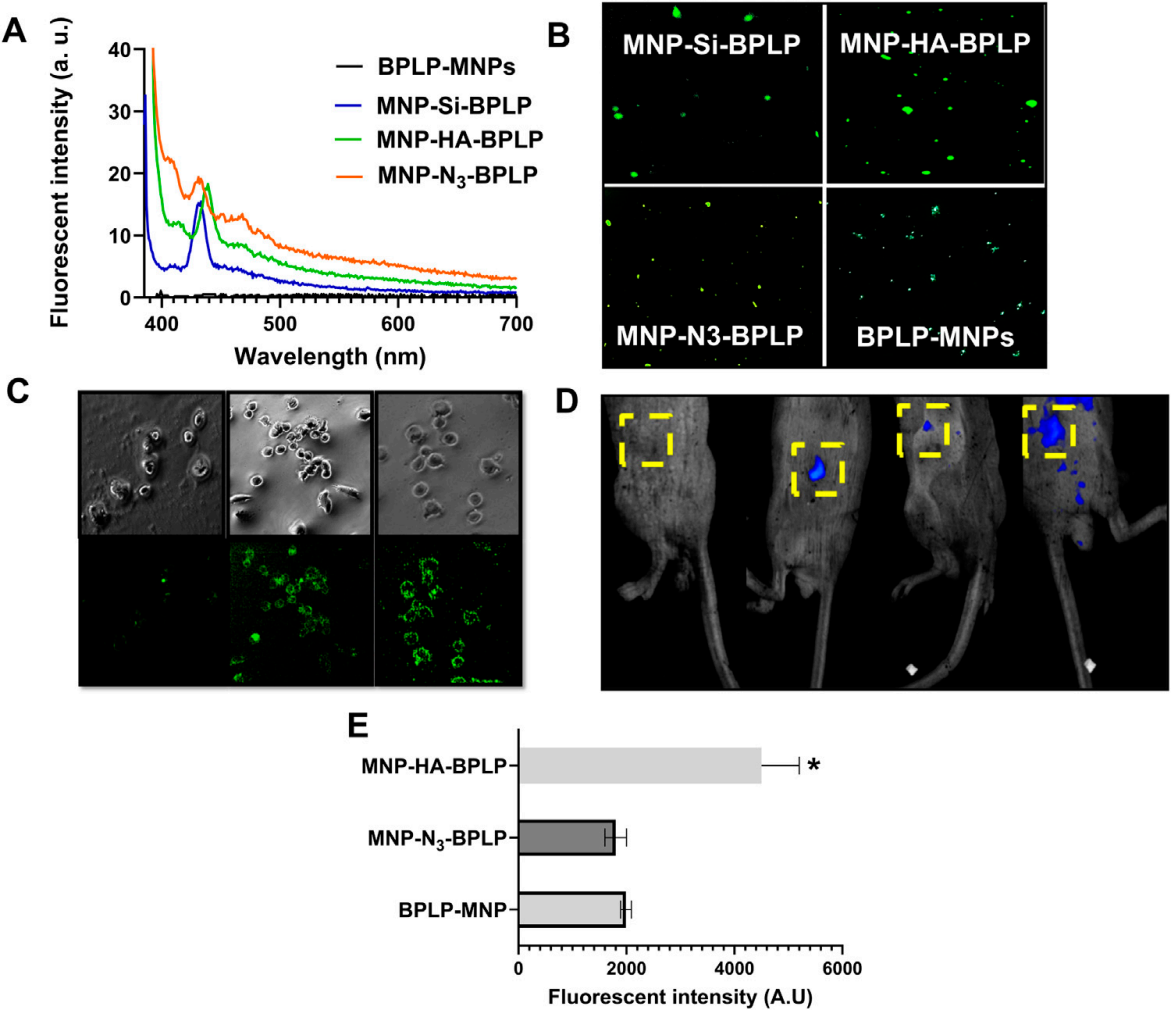
*In vitro* and *in vivo* fluorescence profiles of mDICT NPs. **(A)** Emission spectra of mDICT NPs at a constant excitation wavelength of 375 nm demonstrating BPLP-based fluorescence. **(B)** *In vitro* fluorescence images of mDICT NPs on glass cover slips via Cyto Viva enhanced optical microscope. **(C)** *In vitro* monochrome and FITC-based fluorescent images of PC3 cells with BPLP-MNPs, MNP-HA-BPLP particles, and MNP-N_3_-BPLP particles. **(D)** *In vivo* whole-body images showing fluorescence from (1) tumor only, (2) BPLP-MNPs, (3) MNP-HA-BPLP particles, and (4) MNP-N_3_-BPLP particles. **(E)** Quantified *in vivo* fluorescence intensities of mDICT NPs within subcutaneous tumors.

Our *in vitro* and *in vivo* fluorescent imaging analyses showcase the improved fluorescence of mDICT NPs compared to our previously developed non-modified DICT NPs (Figure 4). The excitation/emission spectra revealed the emission maxima of mDICT NPs to be in the range of 425–440 nm (Figure 4A), consistent with the fluorescence emission spectrum of BPLP nanoparticles of comparable size (λ_max/Em_: 450 nm) (Yang et al., 2009). The mDICT NPs developed using Si and Si-N_3_-based surface modifications had emission maxima between 425 and 430 nm, while the mDICT NPs with HA-based surface modifications showed a slight blue shift in their excitation maxima towards the 450 nm wavelength region (λ_max/Em_: 440 nm).

*In vitro* imaging of mDICT NPs on glass cover slips revealed the presence of robust fluorescent signals from aqueous mDICT nanoparticle suspensions (Figure 4B). DICT NPs (BPLP-MNPs) appeared qualitatively dimmer, indicating a reduction in fluorescence output. Subsequent *in vitro* cellular imaging of NPs incubated with PC3 prostate cancer cells revealed bright fluorescent images of cells outlined with mDICT NPs (Figure 4C). The mDICT NPs developed using HA-based and Si-N_3_-based-MNP surface modifications showcased the best fluorescence contrast in delineating the PC3 tumor cells, while the DICT NPs (BPLP-MNPs) showed a relatively diminished fluorescence-based imaging contrast.

*In vivo* imaging of PC3 subcutaneous tumors treated with intra-tumorally injected NPs demonstrated the ability of these NPs to generate fluorescence signals within tumor tissues (Figure 4D). Quantification of fluorescence intensities from tumor samples revealed that mDICT NPs developed using Si-N_3_-based MNP surface modifications (MNP-N_3_-BPLP) had similar fluorescence outputs as silane-based mDICT NPs (MNP-Si-BPLP). However, interestingly mDICT NPs developed using HA-based surface modifications (MNP-HA-BPLP) had several fold higher fluorescence outputs. The mDICT NPs developed using Si-N_3_-based MNP-surface modifications have the BPLP-alkyne polymer directly conjugated to the MNP surface via copper catalyzed-click chemistry. This bio-orthogonal chemistry may efficiently graft the BPLP polymer onto MNP surfaces, lowering the total absorption cross-section of the magnetic nanoparticle suspension, leading to robust fluorescent signals within tumor cells upon *in vitro* imaging. However, the proximity of the BPLP polymers to MNP surfaces may remain unresolved. Thus, the resulting MNP-based fluorescence quenching could be a factor in the reduced fluorescence output relative to the HA-based mDICT NPs, when imaging tumors *in vivo.*

A study investigating silica-based surface modifications of MNPs reported that the MNP-based quenching effect on Cy5 conjugated dyes varies as a function of distance between Cy5 molecules and the MNP surface, modulated by the thickness of the MNP surface silica coating (Jang et al., 2014). The fluorescence quenching could be controlled by varying the silica coating thickness on the MNP surface. The fluorescence from Cy5 conjugated dyes was heavily quenched (∼97%) when the dye molecules were situated within 10 nm of the MNP surface, while the MNP based quenching effect diminished as the Cy5-MNP distance increased to >50 nm. Our future studies will investigate this spatial separation-based modulation of BPLP’s fluorescence output in the mDICT NPs developed using Si-N_3_-based MNP-surface modifications to investigate the effects of thickness of the Silane-azide layer in fluorescence outputs of the BPLP shell in order to improve imaging properties of these NPs.

Studies on fluorescent MNP systems assembled using oil/water emulsions highlight the importance of controlling the absolute absorption cross-section of MNP suspensions as well as modulating the spatial interaction between fluorophores and the MNP surface (Mandal et al., 2005). For instance, a study on oil/water emulsions of oleic acid coated-MNPs and fluorogenic quantum dots reported on the formation of a fluorescent and strongly magnetic-nanoparticle system. However, the nanoparticle system exhibited reduced fluorescence, wherein, for a given quantum dot size and concentration, the fluorescence loss varied as a function of the MNP content, with a 100 fold reduction as iron oxide content increased from 0% to 51% (Mandal et al., 2005). To resolve fluorescence quenching, the authors emphasized the importance of confining the iron oxide MNPs within the inner volume of emulsions, while localizing the fluorogenic quantum dots at the emulsion-droplet surface. In addition, the increase in absorption cross-section of the MNPs due to the increase in MNP content was credited as the primary factor causing the decrease in fluorescence of this magneto-fluorescent nanoparticle system. The mDICT NPs developed using silane-based MNP surface modifications as well as the HA-based MNP surface modifications (MNP-HA-BPLP) have the MNPs incorporated into a BPLP shell via the double-emulsion process. The creation of such W/O/W emulsions can confine the MNPs in an inner aqueous phase while sequestering the polymeric BPLP in the oil phase. Such spatial compartmentalization may mitigate some of the MNP-based fluorescence quenching.

The mDICT NPs prepared via silane-based MNP surface modifications (MNP-Si-BPLP) indicate that despite such confinement of MNPs within an aqueous phase, mDICT NPs prepared via silane-based MNP surface modifications have reduced fluorescence outputs when imaging tumor cells *in vitro* as well as in subcutaneous tumors *in vivo* (Figures 4C, D) compared to mDICT NPs prepared using HA-based MNP modifications. This could be due to the ineffectiveness of the silane coating in lowering the absolute absorption cross-section of MNPs, which can lead them to strongly absorb the excitation/emission photons, reducing the fluorescence output from the BPLP polymeric shell.

Moreover, the mDICT NPs developed using HA-based MNP surface modifications (MNP-HA-BPLP) had the most robust fluorescence output and were the most optimally configured in attenuating the MNP-mediated fluorescence quenching of the BPLP polymeric shell. This could be due to HA providing an optically inert coat on the MNP surface that does not further lead to absorption of excitation/emitted light in the BPLP polymeric emission wavelength 400–500 nm, thus reducing the absolute absorption cross-section of the mDICT NP liquid suspension. In addition, the HA coating may introduce sufficient interstice between the MNP surfaces and the BPLP polymeric shell that can mitigate MNP-based fluorescence quenching.

### *In Vitro* cyto-compatibility

**FIGURE 5:**
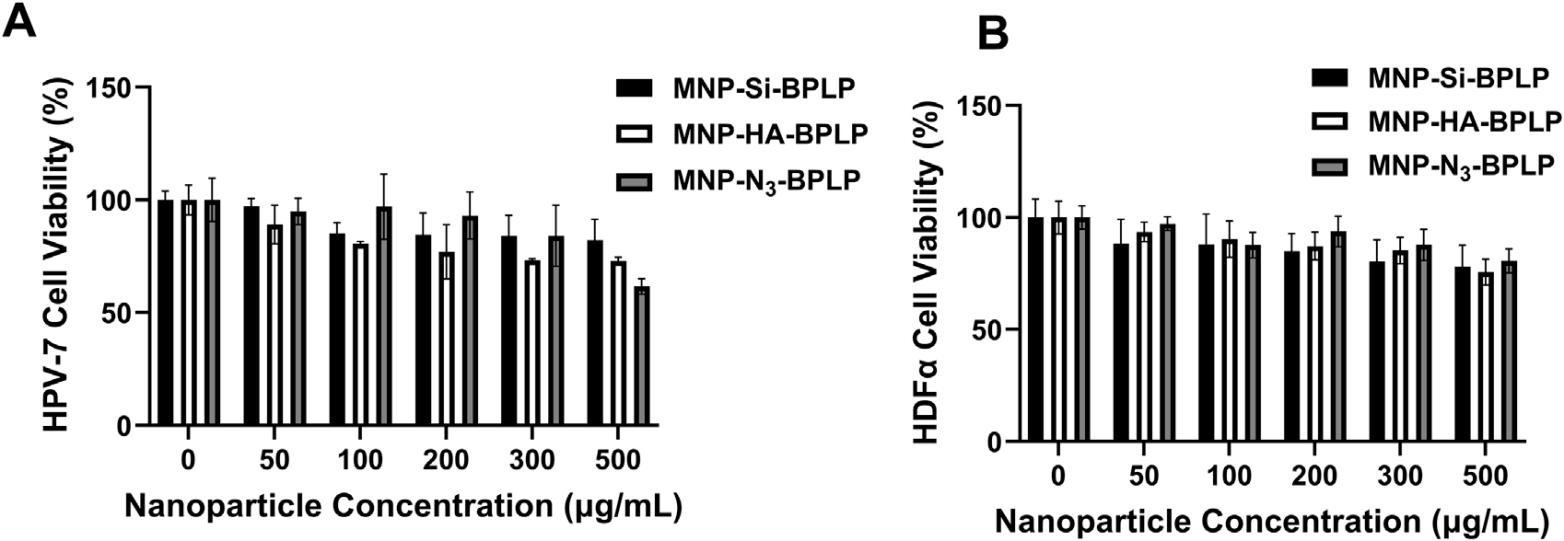
*In vitro* cyto-compatibility and tumor cell uptake of mDICT NPs. **(A)** Human dermal fibroblast (HDFα) cells and **(B)** human prostate epithelial cells (PZ-HPV7) showing acceptable cyto-compatibility after 24 h of nanoparticle exposure.

*In vitro* compatibility analysis of mDICT NPs on normal healthy HDFα and normal human prostate epithelial cells (PZ-HPV-7) revealed that these nanoparticles did not induce any heightened decrease in cellular viability within a 24-h nanoparticle exposure, up to nanoparticle doses of 500 μg/mL (Figures 5A, B). The cellular viability indicated signs of a dose dependent cell survival decrease when mDICT NP treatment concentrations exceeded 100 μg/mL. This was more pronounced in the PZ-HPV-7 cells compared to HDFα and within the nanoparticle groups, the Si and Si-N_3_-based mDICT NPs demonstrated a more enhanced decrease in cellular viabilities, with Si-based mDICT NPs being relatively less compatible.

Although, the *in vitro* cytocompatibility studies demonstrated dose-dependent changes in cell viability, but these effects became apparent only at concentrations exceeding 100 μg/mL. Even at higher concentrations, the overall decreases in viability were not statistically significant, suggesting that the nanoparticles maintain good biocompatibility. These findings indicate that the observed variations in viability do not suggest significant cytotoxicity, further supporting the potential of these nanoparticles for biomedical applications.

Our group has previously reported on the cytocompatibility profiles of BPLPs both *in vitro* when BPLP was co-cultured with 3T3 fibroblasts and *in vivo* upon subcutaneous implantation in rats, showing excellent bio-compatibility profiles (Yang et al., 2009). Biochemically unmodified MNPs have been reported to be toxic to cells and need bio-inert coatings to enable a favorable cyto-compatibility profile. We and others have shown that MNPs coated with various polymers such as PEG, pluronics, and PNIPAAm copolymers have improved cyto-compatibility (Wadajkar et al., 2012; Sundaresan et al., 2014; Lin et al., 2009; Rahimi et al., 2010).

Our results indicate the mDICT NPs to be cyto-compatible for all coatings. This could be due to the inorganic coat of HA on the MNP-core in addition to the BPLP polymeric coat that limits MNP-mediated cellular toxicity, while Si and Si-N_3_-based mDICT NPs with their Si and Si-N_3_ coats limit MNP-core mediated toxicity. In addition, the dose-dependent decrease in cellular viabilities observed in Si and Si-N_3_-based mDICT NPs may be due to increased MNP-mediated oxidative stress at higher densities of nanoparticles in a confined *in vitro* culture medium. In addition, the decrease in cellular viabilities observed in mDICT NPs developed using Si-N_3_-based surface modifications is hypothesized to be attributed to an additional possibility of residual copper ions from copper-catalyzed click chemistry to graft the BPLP shell.

### *In Vitro* cell uptake

**FIGURE 6:**
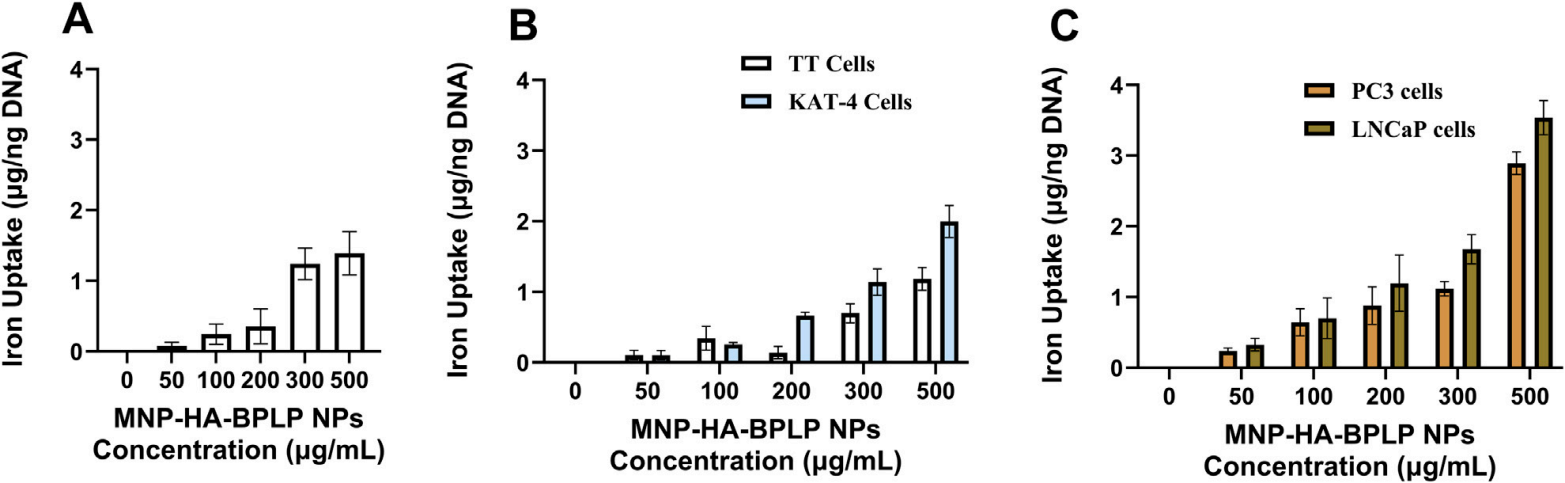
*In vitro* cellular uptake. **(A–C)** Cellular uptake of MNP-HA-BPLP nanoparticles showing dose dependent uptake by **(A)** G-361 skin cancer cells, **(B)** thyroid cancer cells: TT and the KAT-4 cancer cells, and **(C)** PC3 and LNCaP prostate cancer cells.

**FIGURE 7:**
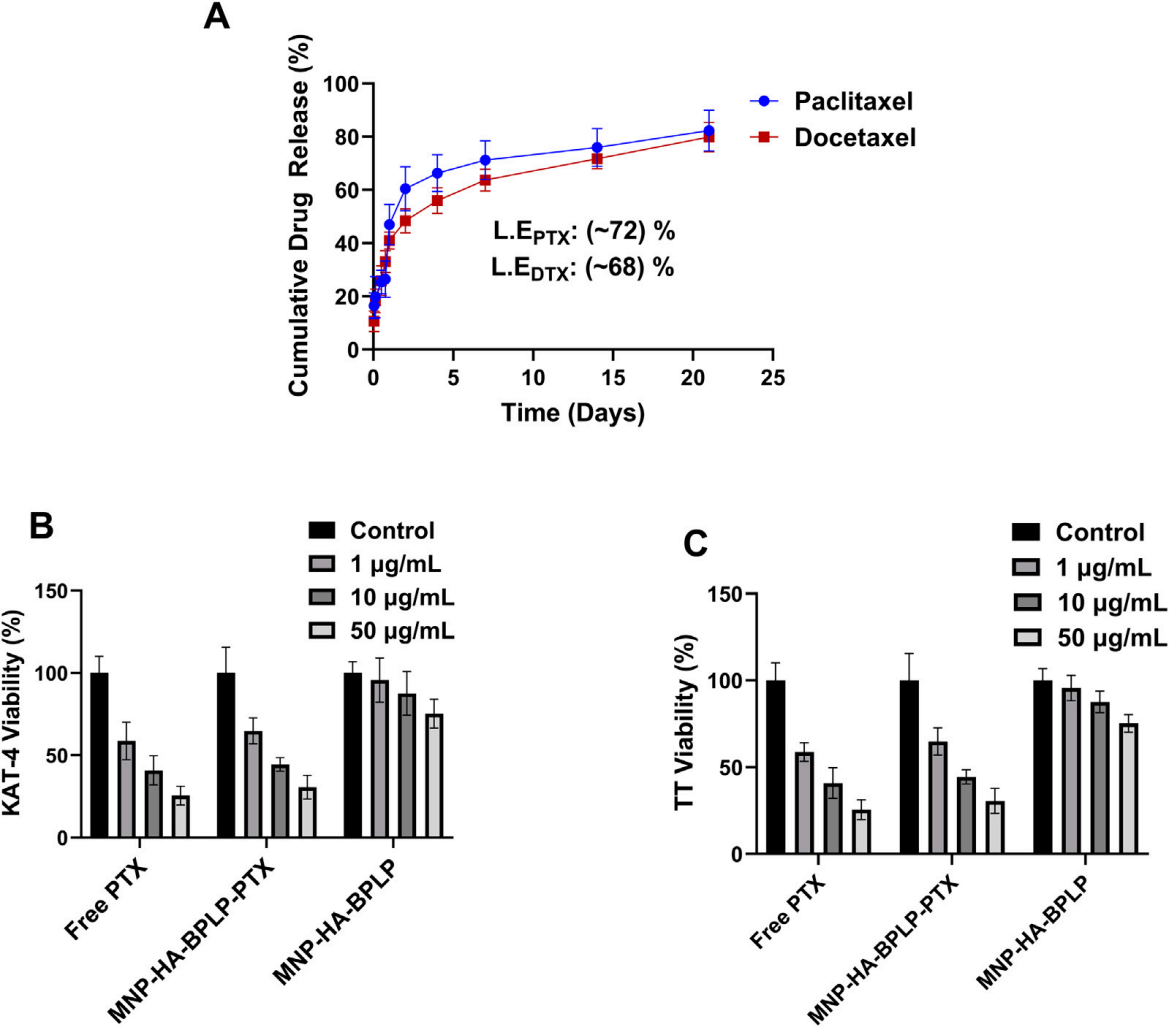
*In-vitro* drug release and tumor-cell cyto-toxicity profiles of HA-based mDICT NPs. **(A)** Paclitaxel and Docetaxel drug release profiles of HA-based mDICT nanoparticles showing sustained drug release over a period of 21 days. **(B–C)** Therapeutic efficacy of drug loaded MNP-HA-BPLP nanoparticles on KAT-4 tumor cells **(B)** and TT-thyroid cancer cells **(C)**.

Cumulatively, our magnetic and fluorescent property characterizations indicate that mDICT NPs developed using HA-based surface modifications (HA-based mDICT NPs: MNP-HA-BPLP) show the most optimal retention of BPLP’s fluorescence, MNP-core’s superparamagnetic properties and T2-weighted contrast based-MR imaging capabilities, alongside superior cellular toxicity profiles relative to Si and Si-N_3_ based-mDICT NPs. Thus, for subsequent *in vitro* proof-of-concept therapeutic studies, we elected to test the *in vitro* cellular uptake and therapeutic efficacies of HA-based mDICT NPs. We analyzed cellular uptake of these NPs on a panel of skin (G361), thyroid (TT, KAT-4), and prostate (PC3, LNCaP) cancer cell lines (Figures 5–7).

Overall, the HA-based mDICT NPs demonstrated dose-dependent cellular uptake in all tested tumor-cell lines (Figures 6A–C), with preferential uptake characteristics within the thyroid cancer cell lines, TT and KAT-4, and also amongst prostate cancer cell lines (PC3, LNCaP). These NPs showed significantly higher uptake in the KAT-4 cell line relative to the thyroid cancer-TT cell line (Figure 6B).

The HA-based mDICT NPs also had significantly higher uptake in LNcaP cells relative to PC3 cells (Figure 6C). The LNCaP cells are positive for a prostate specific membrane antigen (PSMA+) and have a lower metastatic potential relative to PC3 cells (PSMA- and high metastatic potential) (Tolkach et al., 2015; Pogoda et al., 2021). We have observed such differential uptake in our previously developed DICT NPs (BPLP-MNPs) with the variations in uptake profiles between LNCaP and PC3 cell lines being associated with the degree of BPLP-polymer hydrophobicity, wherein greater BPLP hydrophobicity correlated with more favorable cell-biomaterial interactions in LNCaP cells resulting in higher DICT NP uptake relative to PC3 cells (Wadajkar et al., 2012). Since the outer BPLP-shell of HA-based mDICT NPs is identical in biomaterial composition to DICT NPs, we attribute the BPLP-polymeric shell’s hydrophobicity to potential NP-cell lipophilic interactions and higher-uptake of these NPs in LNCaP cells relative to PC3 cells. Such a biomaterial-hydrophobicity driven-cellular-uptake effect has been well recognized for several diverse types of NP systems (Mukadam et al., 2020; Fröhlich, 2012; Sun et al., 2018; Bai et al., 2015; Sasidharan et al., 2011; Li et al., 2008). Given this, we hypothesize the BPLP-polymeric shell’s hydrophobicity to also be one of the underlying causative factors in the differential NP-cell uptake amongst human thyroid cancer cell-TT and the KAT-4 tumor cell lines.

### *In Vitro* drug release and growth reduction profiles of mDICT NPs on cancer cells

The HA-based mDICT NPs were loaded with two model Taxanes: Docetaxel and Paclitaxel with 68% and 71% loading efficiencies and showcased sustained release of both Taxanes in physiologically relevant conditions over a period of 21 days (Figure 7A). The two drug-loaded HA-based mDICT NPs systems showcased an initial burst release of 20% cumulative load released in the first 12 h, followed by a more sustained release pattern, with 50% cumulative drug load released over 2 days and 80% of the cumulative drug load released over 21 days.

We also observed that drug loading into mDICT-NPs does not result in a significant change in nanoparticle size, indicating that the drug incorporation process maintains mDICT NPs structural integrity and colloidal stability. However, in contrast, drug loading via absorption methods in polymeric systems such as thermo-responsive poly (*N*-isopropylacrylamide) has been shown to cause a notable size increase, likely due to polymer swelling and drug-polymer interactions (Chastek et al., 2010).

In subsequent *in vitro* therapeutic efficacy studies, the Paclitaxel-loaded HA-based mDICT NPs (MNP-HA-BPLP-PTX) demonstrated dose-dependent cancer cell killing effects on KAT-4 and TT cell lines demonstrating the therapeutic potential of these mDICT NPs in delivering chemotherapy drugs to cancer cells (Figures 7B,C). Notably, in the 1–50 μg/mL NP concentration range, the HA-based mDICT NPs could affect significant dose-dependent cancer cell killing towards the thyroid cancer TT cells and the KAT-4 cancer cells via cellular uptake and rapid release of Paclitaxel within the 24 h of cell-nanoparticle incubation. The Paclitaxel-loaded HA-based mDICT NPs had the same efficacy in cancer cell growth reduction effects on the tumor cell lines as Paclitaxel alone within the confines of an *in vitro* testing environment within a short period. This bodes well for their probable performance *in vivo*, as Paclitaxel delivery via HA-based mDICT NPs may show higher tumor accumulation via the employment of magnetic targeting, which may lower systemic toxicities associated with the use of Paclitaxel alone.

Overall, the primary objective of this study was to improve the fluorescent properties of mDICT NPs through various surface modification strategies. The cytotoxicity evaluations included in this work were designed as preliminary assessments to demonstrate the drug delivery potential of mDICT NPs rather than a comprehensive toxicity study. As part of future development, long-term cytotoxicity assessments and detailed cellular interaction studies will be conducted to further evaluate nanoparticle behavior in biological systems. These studies will incorporate extended exposure experiments and advanced techniques such as flow cytometry, providing a more in-depth analysis of cellular uptake, apoptosis, and nanoparticle interactions. This will allow for a more complete understanding of mDICT NPs as a potential drug delivery platform.

## Conclusion

This research showcases the use of spatially strategic biochemical surface modifications on MNPs to develop an enhanced magnetic-fluorescent core-shell nanoparticle system that sucessfully overcomes the reduced BPLP fluorescence observed in our previously developed DICT NP system. The diminished fluorescence in DICT NPs was attributed to the quenching effect of the MNP-based core on the fluorescent polymeric shell, as well as the sub-optimal BPLP polymeric coat due to inefficient biochemical grafting on the MNP-based core of DICT NPs. Our findings emphasize the importance of controlling MNP-induced fluoresence quenching of the BPLP polymer by introducing spatial separation between the MNP-core and the BPLP shell through the use of optically inert coatings, and by confining the surface modified-MNPs within the BPLP polymeric shell by using double emulsion methodologies. This biochemical strategy led to the successful development of mDICT NPs, a novel magnetic-fluorescent core-shell nanoparticle system with enhanced fluorescence. This advancement overcomes the fluorescence-based imaging limitations of DICT NPs. The mDICT NPs exhibited enhanced fluorescence outputs, improved *in vitro* and *in vivo* imaging outcomes, cytocompatibility, cellular uptake, and sustained drug release capabilities. These results are promising for future applications in biomedical imaging and theranostic-based image guided tumor diagnosis and drug delivery for cancer therapy.

One minor limitation of our study includes the use of the cell-line KAT-4 as representative of thyroid cancers in our *in vitro* cellular uptake anlysis and final therapeutic efficacy evaluations of Paclitaxel loaded-HA-based mDICT NPs. Through a meticulous literature search, we discovered that the KAT-4 cell line has been identified as a mischaracterized human colorectal cancer cell line: HT-29 (Schweppe et al., 2008; Zhao et al., 2011; Capes-Davis et al., 2010). We acknowledge the use of this globally mischaracterized KAT-4 cell line and its’ employment in our cellular uptake studies with mDICT NPs. However, since our principle focus was on investigating the effect of MNP-surface modifications to mitigate MNP mediated fluorescence quenching in bio-polymeric-fluoroscent-MNP core-shell nanoparticle constructs, the use of this mischaracterized KAT-4 cell line does not change the outcome of our scientific investigation. In addition, the findings of this study remain valid as additional cell lines, including thyroid cancer cell line -TT and prostate cancer cell lines-LnCap, PC3 were also utilized for mDICT nanoparticle testings. The inclusion of multiple solid tumor model cell lines demonstrates the broader applicability of mDICT-NPs beyond a single cancer type. Given that these nanoparticles are designed for general theranostic applications in solid tumors, their characterization across different tumor cell lines supports their potential translational relevance. Our future studies will be focused on clarifying the underlying mechanisms of using hydroxyapatite-based coatings on MNP surfaces, as well as exploring the combination of therapeutic drugs to achieve dual diagnostic and therapy effects indrug delivery systems.

Additionally, the *in vitro* drug release study was conducted at physiological pH (7.4) as a preliminary assessment to establish baseline release kinetics and evaluate the drug delivery potential of mDICT NPs. While this study provides initial insights, further investigations are required to assess drug release under acidic conditions (pH 5.5), which better simulate the tumor microenvironment. As part of future development, we will conduct pH-dependent drug release studies to evaluate the responsiveness of mDICT NPs under tumor-like conditions. This will provide a more comprehensive understanding of their potential as a pH-responsive drug delivery platform.

Finally, the clinical translation of promising nanoparticle-based imaging probes such as mDICT NPs, faces several key challenges despite their potential for cancer detection and image-guided surgery. Regulatory and safety concerns remain significant, as FDA approval requires extensive safety evaluations to minimize off-target toxicity. Pharmacokinetics and biodistribution challenges arise from the need to ensure efficient clearance, as larger nanoparticles (>10 nm) tend to accumulate in the reticuloendothelial system (RES), potentially leading to adverse effects. Achieving effective tumor targeting is also complex, as the enhanced permeability and retention (EPR) effect is not always reliable, and receptor variability can impact the success of actively targeted nanoparticles. Additionally, imaging limitations pose difficulties in distinguishing specific tumor targeting from nonspecific uptake, especially in early clinical trials where tumor biopsies for histological validation may be restricted. Furthermore, manufacturing and scalability issues complicate the production of consistent nanoparticle formulations with precise size, charge, and surface chemistry, impacting long-term stability and clinical application. Lastly, clinical trial design and patient recruitment present obstacles, as early-phase studies primarily focus on safety and pharmacokinetics, limiting optimization for tumor detection and treatment efficacy. Overcoming these challenges will be essential to fully realize the potential of nanoparticle-based probes such as mDICT NPs in clinical oncology.

## Data Availability

The original contributions presented in the study are included in the article/supplementary material, further inquiries can be directed to the corresponding authors.
